# Identification of Interactions between Sindbis Virus Capsid Protein and Cytoplasmic vRNA as Novel Virulence Determinants

**DOI:** 10.1371/journal.ppat.1006473

**Published:** 2017-06-29

**Authors:** Kevin J. Sokoloski, Lauren M. Nease, Nicholas A. May, Natasha N. Gebhart, Claire E. Jones, Thomas E. Morrison, Richard W. Hardy

**Affiliations:** 1 Department of Microbiology and Immunology, and the Center for Predictive Medicine for Biodefense and Emerging Infectious Diseases, University of Louisville School of Medicine, Louisville KY, United States of America; 2 Department of Biology, College of Arts and Sciences, Indiana University, Bloomington IN, United States of America; 3 Department of Immunology and Microbiology, University of Colorado School of Medicine, Aurora, CO, United States of America; University of Pennsylvania School of Medicine, UNITED STATES

## Abstract

Alphaviruses are arthropod-borne viruses that represent a significant threat to public health at a global level. While the formation of alphaviral nucleocapsid cores, consisting of cargo nucleic acid and the viral capsid protein, is an essential molecular process of infection, the precise interactions between the two partners are ill-defined. A CLIP-seq approach was used to screen for candidate sites of interaction between the viral Capsid protein and genomic RNA of Sindbis virus (SINV), a model alphavirus. The data presented in this report indicates that the SINV capsid protein binds to specific viral RNA sequences in the cytoplasm of infected cells, but its interaction with genomic RNA in mature extracellular viral particles is largely non-specific in terms of nucleotide sequence. Mutational analyses of the cytoplasmic viral RNA-capsid interaction sites revealed a functional role for capsid binding early in infection. Interaction site mutants exhibited decreased viral growth kinetics; however, this defect was not a function of decreased particle production. Rather mutation of the cytoplasmic capsid-RNA interaction sites negatively affected the functional capacity of the incoming viral genomic RNAs leading to decreased infectivity. Furthermore, cytoplasmic capsid interaction site mutants are attenuated in a murine model of neurotropic alphavirus infection. Collectively, the findings of this study indicate that the identified cytoplasmic interactions of the viral capsid protein and genomic RNA, while not essential for particle formation, are necessary for genomic RNA function early during infection. This previously unappreciated role of capsid protein during the alphaviral replication cycle also constitutes a novel virulence determinant.

## Author summary

Alphaviruses can cause significant disease in infected individuals; however, our understanding of the molecular interactions that enable infection and contribute to the development of disease is limited. The work detailed in this manuscript characterizes the interaction of a viral RNA-binding protein, Capsid, with the viral genomic RNA. Importantly, these interactions were found to be at specific sites on the genome but not essential for virus assembly. Mutation of the capsid / RNA interaction sites decreased the replication of the virus and the severity of disease in a mouse model of infection. Taken together, these findings identify a previously undiscovered determinant of disease severity, and provide a potential basis for the development of new vaccines.

## Introduction

Alphaviruses are positive-sense RNA viruses that exhibit a broad host range; and as evidenced by the emergence of chikungunya virus (CHIKV), represent a significant burden on the public health systems of developed and underdeveloped communities [1–9]. Alphaviral disease is broadly classified based on the symptomology associated with clinical infection. Arthritogenic alphavirus infections, such as those involving CHIKV, Ross River virus (RRV), and Sindbis virus (SINV), induce debilitating arthritis in infected individuals [10]. In contrast to the arthritogenic alphaviruses, infection with encephalitic alphaviruses may result in severe neurologic disease and significant mortality, primarily in young children [11, 12]. Regardless of disease symptomology the members of the genus Alphavirus exhibit highly similar single-cell replication cycles [10, 13].

Mature infectious alphavirus particles are approximately 70 nm in diameter, and consist of two concentric protein shells divided by a host derived lipid envelope [14, 15]. The arrangement of the outer protein shell, consisting of the viral glycoproteins E1 and E2, and the inner protein shell, comprised of capsid protein, are arranged with icosahedral symmetry [14, 15]. The innermost icosahedral structure is the nucleocapsid core, which consists of the viral capsid protein and the RNA cargo. However, the assembly of mature alphavirus particles is a highly selective process as, to date, many characterizations of alphaviral particles have agreed that the viral genomic RNA is the predominant RNA molecule within the nucleocapsid core [16–20]. Several studies have identified a region of the genomic RNA associated with the selective packaging of the viral genome during the assembly of infectious particles. This element, termed the Packaging Signal, consists of a highly-structured region found within the open reading frame of the nonstructural polyprotein [16–18, 21–23]. While these studies provided an excellent definition of the cis-acting elements involved in the selection of the cargo RNA, they did not identify nor describe the interaction between the capsid protein and the viral RNA cargo [18]. The interactions between the alphaviral capsid protein and the viral genomic RNA is an under characterized, yet vitally important, RNA:protein interaction. Alphaviral *in vitro* assembly systems have largely indicated that the assembly of Capsid:nucleic acid complexes is a nonspecific process [24–28]. However it should be noted that the direct interaction between the viral capsid protein and viral RNA has not been exhaustively characterized in the cytoplasm of infected cells, or in mature viral particles leaving our understanding of the molecular interactions between these essential components of the virus incomplete.

This report details our efforts to identify and characterize the sites of interaction between the viral capsid protein and the genomic RNA using the model alphavirus Sindbis virus (SINV). A CLIP-seq approach was used to screen for potential sites of Capsid:RNA (C:R) interaction within Sindbis virus (SINV) particles and cytoplasmic nucleocapsid complexes. Interestingly, while the C:R interactions of purified viral particles are relatively evenly distributed across the genome, the C:R interactions in cytoplasmic RNA:protein complexes exhibit a discrete binding profile. Further characterization of these candidate C:R interaction sites showed a decrease in capsid interaction when the sites were mutated, and indicated that the sites are essential for efficient viral growth as mutation of individual C:R interaction sites significantly reduces the infectivity of the mature viral particles. Further analysis indicated that the C:R interactions are necessary at an early stage of virus infection and stabilize incoming viral genomic RNA. Moreover, the cytoplasmic C:R interaction sites represent a series of novel pathogenicity determinants as C:R interaction site mutants are significantly attenuated in a neurotropic mouse model of infection. Collectively, the results presented indicate a new role for capsid protein during the viral replication cycle and identify a novel determinant of viral pathogenesis expanding our understanding of how Capsid:RNA interactions regulate viral infection beyond particle assembly.

## Results

### Identification of Sindbis virus capsid:RNA (C:R) interaction sites

**Fig 1:**
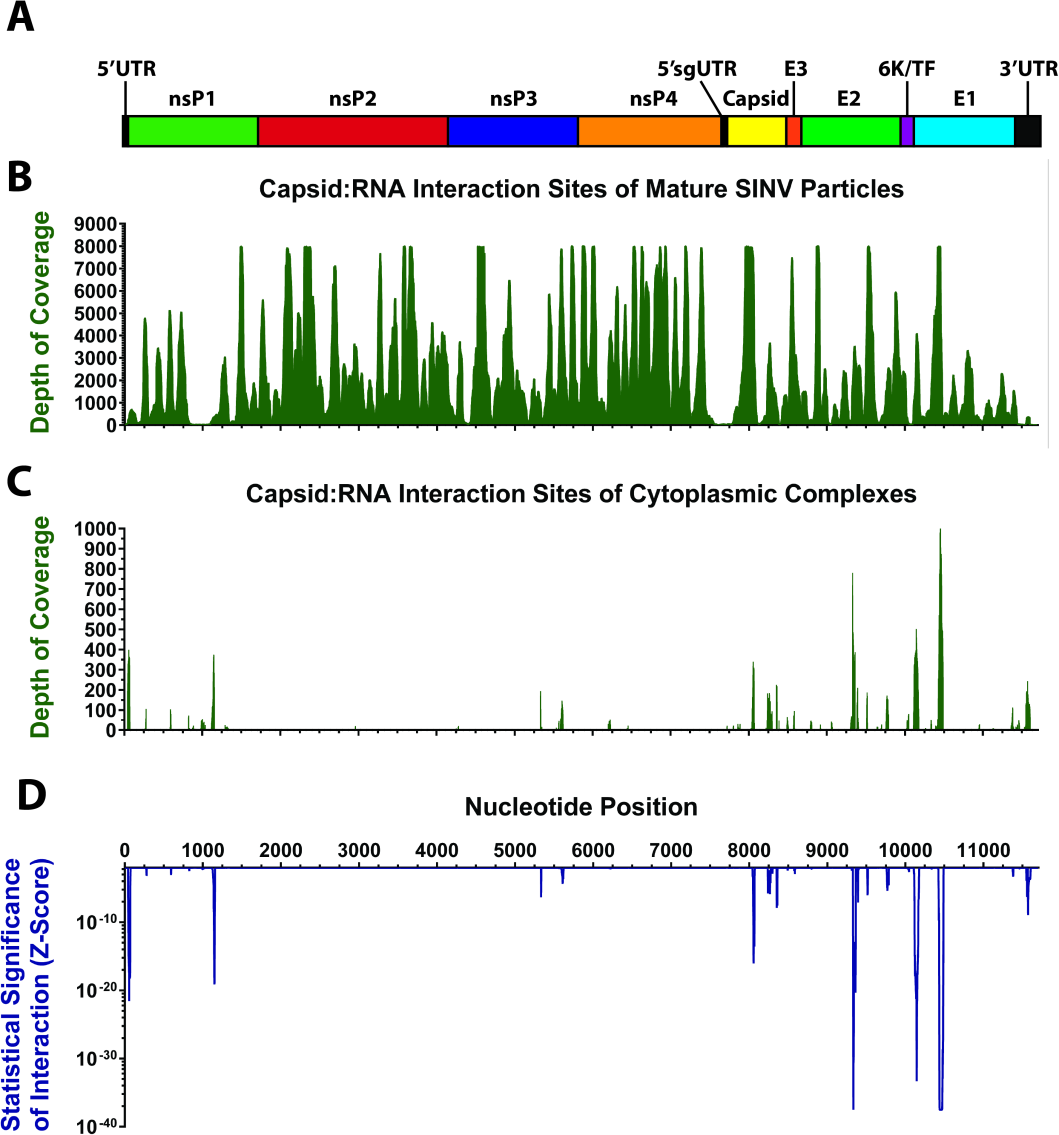
Sindbis virus capsid protein associates with viral RNA in the cytoplasm of infected cells at discrete interaction sites. **A**) A schematic drawing of the genomic RNA of SINV. Protein coding and noncoding regions are labeled. For reference the graphs in panels B through D are aligned with this schematic. **B**) Coverage of anti-Capsid CLIP-seq libraries derived from purified, mature infectious viral particles. Depth of coverage is represented by the y-axis, with the x-axis representing nucleotide position. **C**) Depth of coverage for anti-Capsid libraries derived from cytoplasmic fractions. Plotted on the y-axis is the depth of coverage of the anti-Capsid libraries following subtractive analysis, with the x-axis representing nucleotide position. **D**) Statistical analysis of the data presented in panel C, with the y-axis indicating the Z-score of all represented sequences. The x-axis represents the nucleotide position, with all panels above being aligned with the indicated nucleotide numbering intervals.

To identify candidate site(s) of interaction between the viral genomic RNA and the capsid protein we utilized a CLIP-seq method to develop cDNA libraries of C:R interactions [29–31]. Briefly, either purified viral particles or infected tissue culture cells were irradiated with short-wavelength UV radiation to form covalently cross-linked RNA:protein complexes. The RNA components of cross-linked complexes were then fragmented via RNase digestion. The fragmented C:R complexes were then selectively immunoprecipitated from the lysates using anti-capsid polyclonal antibodies (Supporting Information, S1 Fig). From the immunoprecipitated materials, cDNA libraries were generated using an adaptor ligation process and deep sequenced. In addition to anti-Capsid CLIP-seq libraries, derived from purified virions and infected cells, nonspecific control libraries were also developed. For all cDNA libraries the sequencing data were aligned and clustered to a reference genome (Fig 1A, and Supporting Information S1 Data), consisting of the input SINV Toto1101 parental sequence.

#### The C:R interactions in mature virions are dispersed and nonspecific

CLIP-seq analysis of purified SINV particles indicates that the contacts between the viral capsid proteins and the genomic RNAs are extensive, and likely nonspecific in nature. As shown in Fig 1B, the interactions between the encapsidated genomic RNA cargo and the viral capsid protein in purified viral particles are widely dispersed across the genome. Nonetheless, two regions of the genome exhibited decreased C:R interaction relative to the entirety of the genomic RNA. Interestingly, one of these sites corresponds to the previously identified packaging signal [18]. The second region of low coverage corresponds to the subgenomic promoter and the nucleotides corresponding to the 5’UTR of the subgenomic RNA [10, 13].

#### The C:R interactions of cytoplasmic nucleocapsid complexes exhibit specificity

In contrast to the sequence coverage observed with purified virions, C:R complexes derived from infected cell lysates exhibit remarkable specificity. Co-precipitation of viral RNA and capsid protein was dependent on crosslinking and the use of antibodies specific for capsid (S1 Fig). Subtractive analysis of nonspecific control and anti-capsid cDNA library sequencing data revealed several discrete regions of significant enrichment in the anti-capsid library (Fig 1C). The most highly enriched regions correspond to the coding sequence of the structural ORF, in particular the E2 and E1 genes. Nonetheless, several minor peaks are found in other regions of the genomic RNA, including the 5’ and 3’ UTRs and the nonstructural and structural coding regions. Interestingly, these peaks do not correlate with the previously described Packaging Signal or other known cis-acting features of the viral RNA [13, 18]. To prioritize which peaks would be further characterized we utilized a statistical method to examine the relative enrichment of individual sequences against the entire subtractive data set. As depicted in Fig 1D, statistical analysis of these data indicated that several of the regions with substantial depth-of-coverage were statistically enriched (as determined by Z-score) relative to other sequence clusters. Collectively, these data indicate that the intracellular C:R interactions occur at discrete interaction sites on the viral genome and that the C:R interaction sites found within the structural ORF are the most significantly enriched.

**Fig 2:**
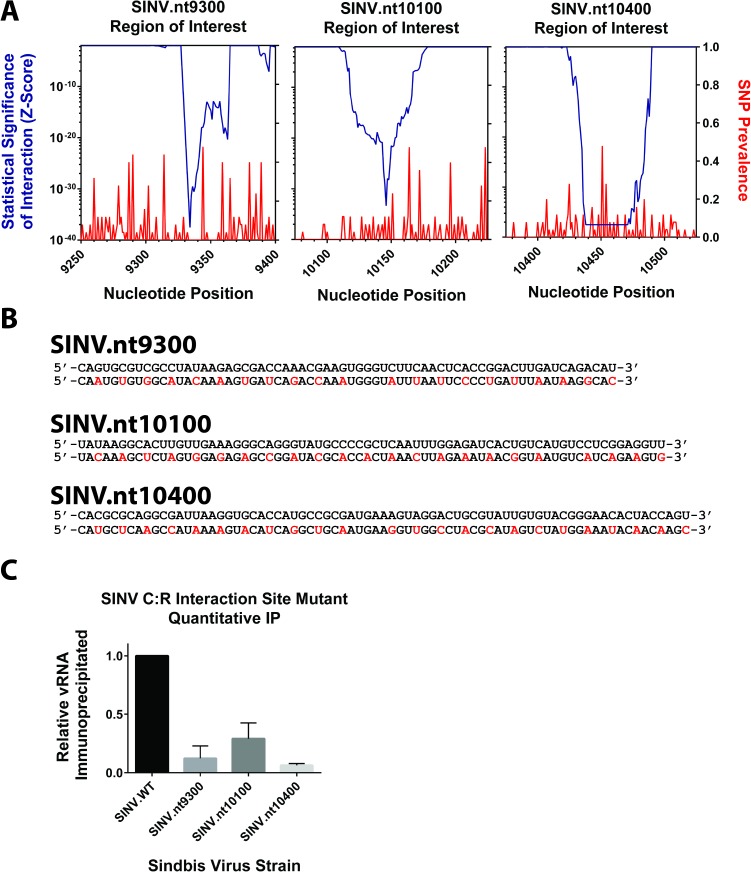
Analysis and mutation of the SINV C:R interaction sites. A) Magnified regions of the viral genomic RNA with special emphasis on the individual C:R interaction sites prioritized during this study. Plotted on the left y-axis are the Z-scores previously reported in Fig 1. The right y-axis indicates the prevalence of single nucleotide polymorphisms (SNPS) across a curated set of SINV genomic RNAs; higher values indicate increased base identity variation at a particular nucleotide but are not informative as to relative base conservation. Nucleotide position is reported on the x-axis. B) The individual nucleotide sequences of the C:R interaction sites are described in regards to parental sequence (top) and mutant sequence (bottom). The mutated nucleotides are highlighted in red. C) Quantitative analysis of the individual C:R interaction sites following mutation indicates that Capsid:RNA binding is significantly reduced relative to parental virus. Data shown is the mean of three independent biological replicates, with the error bar representing the standard deviation of the mean.

As described earlier, several of the intracellular C:R interaction sites were statistically enriched relative to others. These sites, henceforth referred to as nt9300, nt10100, and nt10400 based on their approximate nucleotide locations in the viral genome, were prioritized for further characterization. Initially, we sought to determine if the intracellular C:R interaction sites exhibited similar RNA secondary structures, or primary sequence motifs. Bioinformatic analysis of the nt9300, nt10100, and the nt10400 regions (including up to 100 flanking nucleotides) using mFold failed to identify any RNA secondary structures that were energetically favorable [32]. Similarly, analysis of the nt9300, nt10100, and nt10400 C:R interaction sites did not reveal primary sequence motifs, and did not overlap with known subgenomic promoter elements [33]. Nonetheless, bioinformatic analysis of the individual intracellular C:R interaction sites indicates that the regions identified as highly enriched C:R interaction sites are well conserved across SINV strains. As shown in Fig 2A, the prevalence of single nucleotide polymorphisms, as determined by the sequence alignment of 25 independent SINV strains (Supporting Information S1 Table), indicated that the regions of interest exhibited few SNPs. Numerous residues within the C:R interaction sites were absolutely conserved; furthermore, the overall incidence of SNPs was on average comparable to or below neighboring sequences that were not identified as C:R interaction sites.

Mutation of the candidate interaction sites reduced capsid binding confirming the relevance of the RNA sequence for the interaction with capsid. Analysis of the individual C:R interaction site mutants by quantitative immunoprecipitation following cross-linking indicated that mutation of each of the candidate C:R interaction sites reduced capsid binding. As shown in Fig 2C, the retention of the individual C:R interaction sites following capsid immunoprecipitation was significantly reduced relative to parental virus for each of the C:R interaction sites. These data support the identification of the C:R interaction sites as bona fide capsid:RNA interaction sites and indicate that the mutational approach is capable of reducing capsid interaction at these specific sites.

### Mutation of SINV C:R interaction sites decreases viral growth kinetics

Following the identification of the cytoplasmic SINV C:R interaction sites we next sought to determine their biological importance to viral infection. To this end we developed a series of C:R interaction site mutants. Due to the C:R interaction sites being present in open reading frames it was necessary to maintain amino acid identity during mutation. Therefore, mutations were limited to substitutions of the wobble-base position of each codon (Fig 2B). Importantly, the mutants were designed to maintain codon usage rarity to prevent changes in translational efficiency. In some instances no alternative codons were available (such as for methionine), and as a result the corresponding codons were not altered.

**Fig 3:**
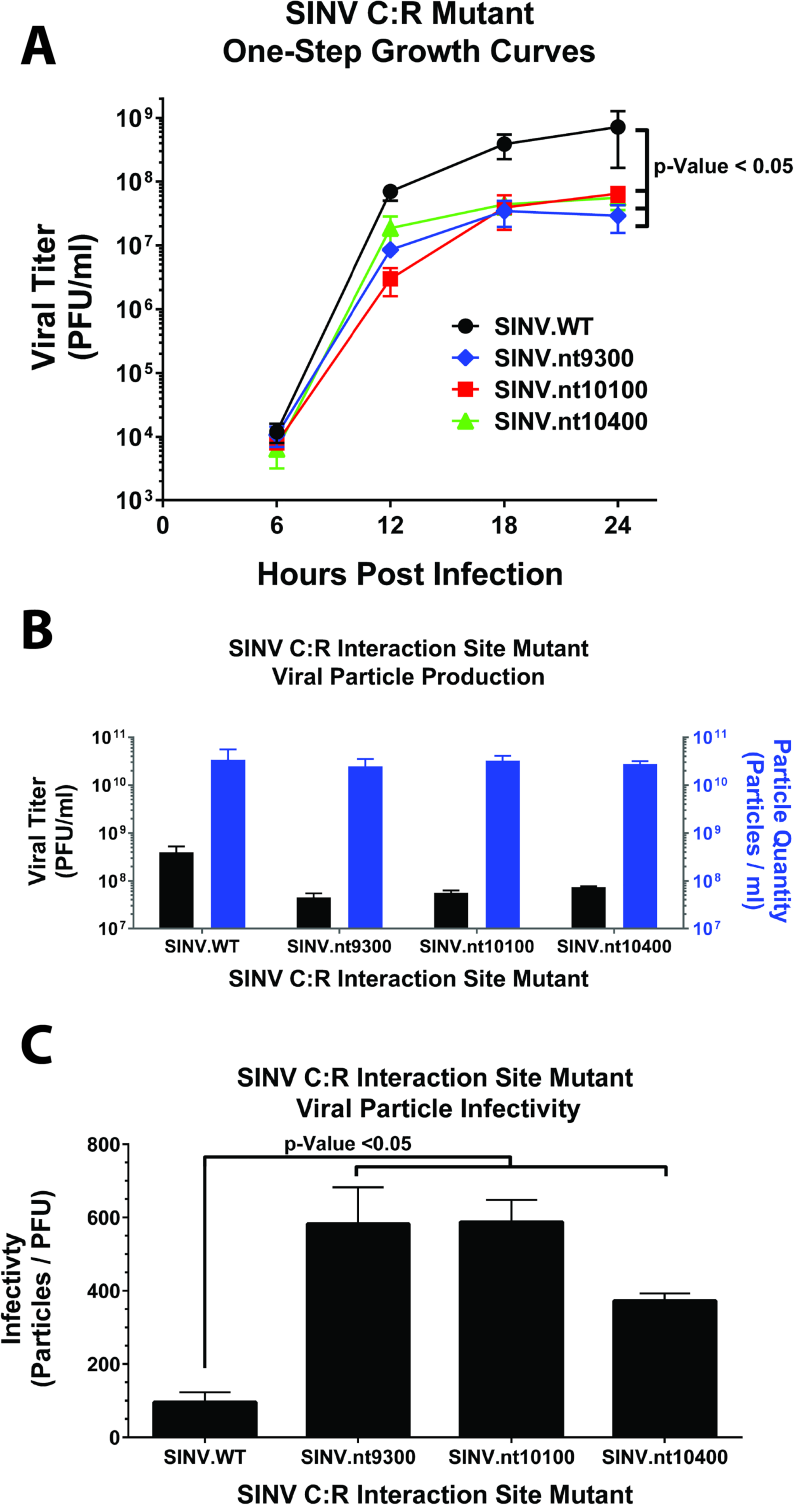
Analysis of SINV C:R interaction site mutant growth kinetics and infectivity. **A**) The one-step growth kinetics of the individual SINV C:R interaction site mutants and parental wild type virus were assessed in HEK293 cells. **B**) Quantitative analysis of the SINV C:R interaction site mutants and parental wild type virus in regards to the number of infectious units (left y-axis), and the total number of viral particles produced as measured by qRT-PCR (right y-axis). **C**) The infectivity of the individual SINV C:R interaction site mutants and parental wild type virus as reported as the ratio of total particles per infectious unit as determined using BHK-21 cells. All quantitative data in this figure represents the mean of three independent biological replicates, the error bar representing the standard deviation of the mean. Statistical significance, as indicated on the individual panels above, are the p-Values obtained from Student’s t-test.

The one-step growth kinetics of the C:R interaction site mutants were characterized in mammalian tissue culture cells. As shown in Fig 3A, mutation of each of the individual SINV C:R interaction sites significantly reduced the number of infectious particles produced during infection. On average, a reduction of ~15-fold in the yields of infectious virus at 24 hpi was detected for the C:R nt9300, nt10100, and nt10400, compared with wildtype SINV.

Since a potential consequence of disrupting the interactions between the viral capsid protein and the genomic RNA could be a reduced production of viral particles, we next sought to determine if particle production was negatively affected in C:R interaction mutant viruses. Interestingly, as shown in Fig 3B, the total number of viral particles produced (as measured by genome equivalents per unit volume) by both wildtype SINV and C:R interaction site mutants were numerically, and statistically, equivalent. Therefore, the production of viral particles was not perturbed by mutation of the C:R interaction site mutations. However, the capacity of the SINV C:R interaction site mutant viruses to successfully initiate infection was significantly reduced as demonstrated by their relative infectivity (as measured by the ratio of particles to infectious units) depicted in Fig 3C.

**Fig 4:**
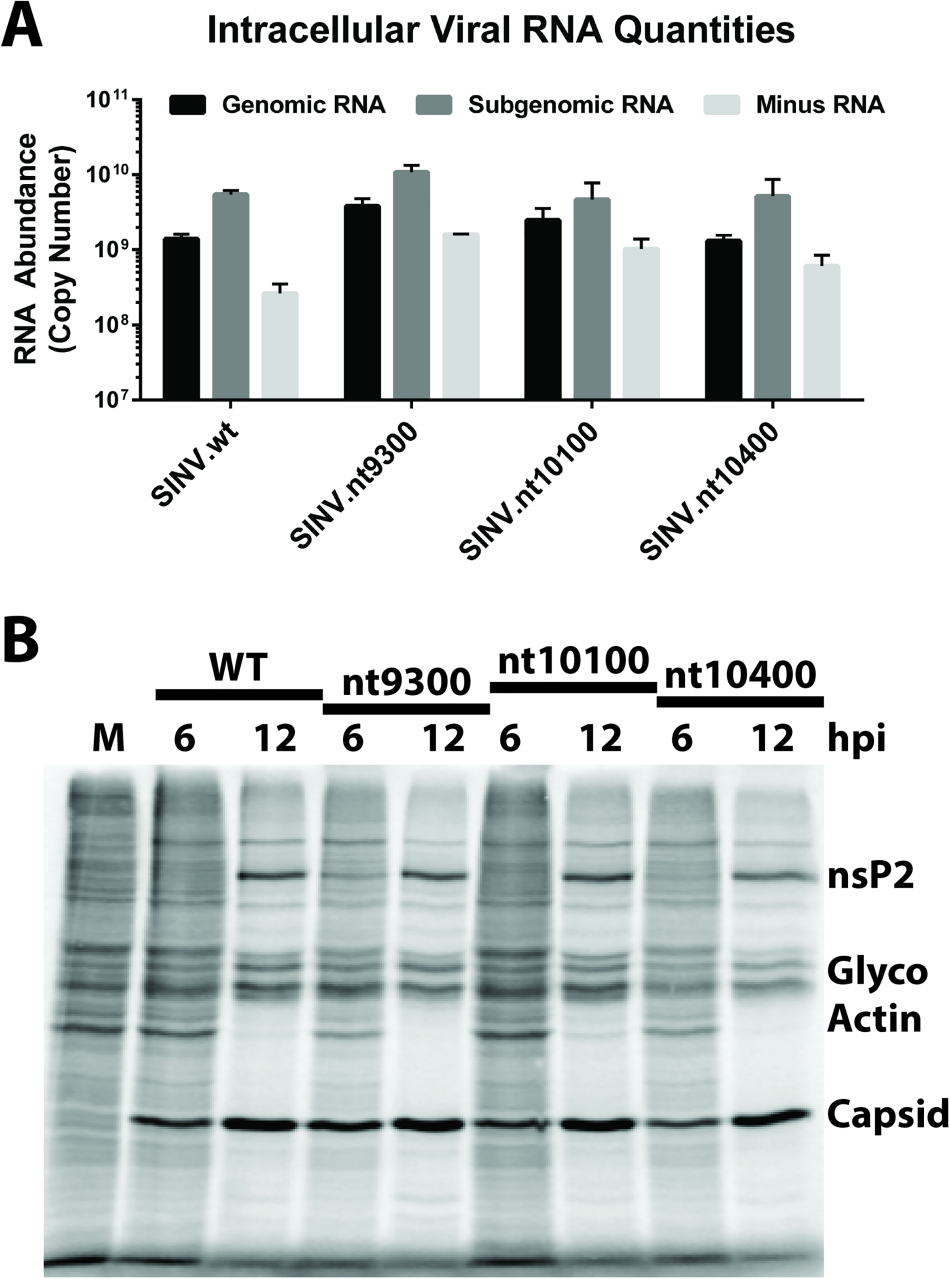
Assessment of SINV C:R interaction site mutant RNA synthesis and gene expression. **A**) Quantitative analysis of the three viral RNA species generated during SINV infection of HEK293 cells at 12-hours post infection at an MOI of 10 infectious units per cell. The copy numbers of the Genomic, Subgenomic, and Minus strand RNAs were quantified using qRT-PCR. Data in this panel represents the mean of three independent biological replicates, the error bar representing the standard deviation of the mean. **B**) Metabolic labeling of infected HEK293 cells at the indicated times. Infected cells were metabolically labeled for a period of two hours immediately preceding the time indicated in the figure. The migratory positions of nsP2, Capsid, and the viral glycoproteins; and host Actin, are indicated to the right of the panel. Data shown is representative of several independent biological replicates and technical replicates.

To extend our understanding of the molecular impact of C:R interaction site mutation we assayed viral RNA synthesis and viral gene expression. As demonstrated in Fig 4A mutation of the C:R interaction sites did not significantly alter either the accumulation, or relative ratios of the individual SINV RNA species. For each of the C:R interaction site mutants, the accumulation of the minus-strand RNA was slightly increased relative to wildtype levels. While this was consistent over multiple biological replicates, the observed differences in minus-strand accumulation are relatively minor. Analysis of viral gene expression by metabolic labeling of HEK293 cells at 6 and 12 hours post infection indicates that viral gene expression is similarly unperturbed by C:R interaction site mutation (Fig 4B). Additionally, host cell shutoff, as indicated by the relative intensity of the actin band, is equivalent at 6 and 12 hpi for the C:R interaction site mutants and wildtype SINV at equal MOI in terms of infectious units per cell.

The data reported above indicates that the C:R interaction sites identified by CLIP-seq analysis of cytoplasmic complexes are not directly involved with nucleocapsid assembly and particle release. Moreover, the lack of an apparent defect in viral RNA synthesis or structural gene expression indicates that the SINV gene products are functioning normally, and that the C:R interaction site mutants are, late during infection, equivalent to wildtype virus. However the infectivity of SINV C:R interaction site mutants is significantly diminished relative to parental virus. Hence, these data suggest that mutation of the C:R interaction sites negatively affects an early event of the viral lifecycle prior to viral RNA synthesis.

### Capsid:RNA interactions are vital early during infection

The data acquired indicted that while the C:R mutants did not inhibit particle production the infectivity of the particles was decreased. However, once infection was established the synthesis of viral RNAs and viral gene expression was unaffected. These data implied that the C:R mutations disrupted particle infectivity at an early stage of infection and that the cytoplasmic C:R interactions were less essential at later stages of infection. Previously, we demonstrated that alphaviral infectivity is largely determined by the functional capacity and stability of the genomic RNA immediately following viral entry [34]. On this basis we determined the genomic RNA half-lives for each of the individual C:R interaction site mutants at very early times post-infection during a synchronized infection of HEK293 cells.

**Fig 5:**
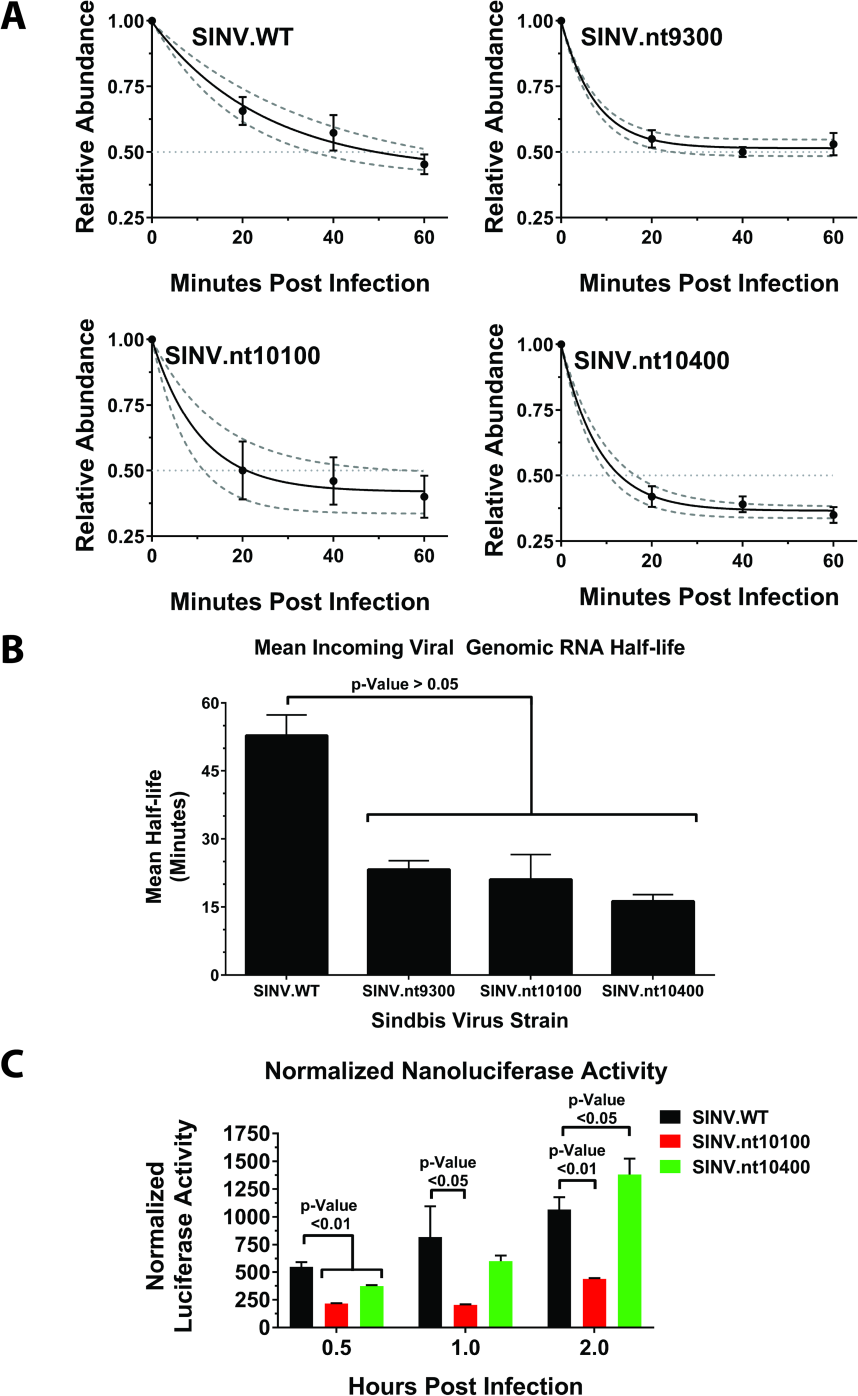
Mutation of the SINV C:R interaction sites negatively affects incoming viral genomic RNA function. **A**) The RNA decay profiles of the incoming viral genomic RNAs of the individual SINV C:R interaction site mutants and parental wild type virus were determined by qRT-PCR analysis as described in the materials and methods. Plotted is the relative abundance of the incoming viral genomic RNAs (y-axis) with regards to time (x-axis). Regression analysis was utilized to determine the RNA decay profile (as shown with the solid line) and the dashed lines represent the 95% confidence intervals of the aforementioned regression. **B**) The half-lives of the individual genomic RNAs as determined using the calculations reported in Dolken et al., as determined by the first point at which the relative abundance has reached 0.5. C) The levels of Nanoluciferase activity for wild type parental virus and the nt10100 and nt10400 C:R interaction site mutants were determined as reported in the materials and methods at the indicated times post infection. All quantitative data in this figure represents the mean of at least three independent biological replicates. Comparative analysis was performed using variable bootstrapping, as described in the materials and methods, with the error bar representing the standard deviation of the mean. Statistical significance, as indicated on the individual panels above, are the p-Values obtained from Student’s t-test.

To this end, HEK293 cells cultured in the presence of the uridine analogue 4-thiouridine (4SU) were infected at an MOI of 5 infectious units per cell at 4°C to allow for viral adsorption but not entry. After the initial adsorption period, the cell monolayers were extensively washed to remove unbound particles and pre-warmed media containing 4SU was added to release the block to viral entry. At the indicated times post-infection the total cellular RNA was harvested and the incoming genomic RNAs purified from the nascent transcribed RNAs. The viral genomic RNAs were then quantified using qRT-PCR as described in the materials and methods. As shown in Fig 5A, the stability of the C:R interaction site mutant viral RNAs was decreased compared with the stability of wildtype SINV RNA. Importantly, the RNA half-life observed during these studies is very similar to that previously reported [34]. However, and interestingly, while wildtype SINV exhibits a steady monophasic decay profile, the individual C:R interaction site mutants exhibit multi-phasic decay, with an initial period of notable instability followed by a prolonged period of stability. Calculation of the individual half-lives for each of the viruses used in this study indicates that mutation of C:R interaction sites destabilizes the incoming viral genomic RNAs significantly, decreasing the mean half-life by 2-fold on average (Fig 5B).

Collectively, these data indicate that mutation of the individual C:R interaction sites results in destabilization of the incoming genomic RNAs early during infection. Since efficient function of the incoming viral genomic RNA is essential to the establishment of viral infection, it is, a priori, understandable that failure of the genomic RNA early in infection, such as RNA instability, would result in reduced viral infectivity.

Aside from persisting in the host cytoplasm, another important molecular function of the incoming genomic RNA is to act as an mRNA for the synthesis of the viral replication machinery. Moreover, since the translational capacity of a transcript often correlates with the relative stability of an RNA it is likely that the earliest translation events of C:R interaction site mutants are also perturbed [35]. As such, we next sought to determine if the translational activity of the incoming viral genomic RNAs differed amongst wild type and C:R interaction site mutant viruses. To this end we utilized a reporter SINV strain that expresses Nanoluciferase internal to the nsP3 protein. This construct enables highly sensitive detection of gene expression early during infection, similar to previously described. To assess the translational capacity of the C:R interaction site mutants we developed a series of individual C:R interaction site mutants in the nsP3 Nanoluciferase reporter backbone. Unfortunately, despite many attempts we were unable to recover SINV nsp3.nanoluc nt9300 mutant virus as the resulting mutant was so severely attenuated.

The translational activity of the parental wild type SINV nsP3 Nanoluciferase construct and the nt10100 and nt10400 C:R interaction site mutants was assessed in HEK293 cells. Briefly, HEK293 cells were infected at an MOI of 10 infectious units per cell. After the removal of unbound viral particles the cells were harvested at 30 min, 1 hr, and 2 hrs post infection and assayed for nanoluciferase activity. As shown in Fig 5C, wild type parental virus exhibited steady expression during early infection. However, both nt10100 and nt10400 exhibited significantly reduced translational activity early during infection. Indeed, at 30-minutes post infection the normalized nanoluciferase activity was reduced 2.5-fold and 1.5-fold for the nt10100 and nt10400 mutants, respectively. At 1-hour post-infection the nanoluciferase activity of the nt10100 mutant was further reduced, approximately 4-fold relative to wild type levels. However, at the same time point, the nt10400 mutant exhibited an increase in nanoluciferase activity to more or less wild type levels, indicating that the defect imparted by the nt10400 mutant is short lived. Moreover, the nanoluciferase expression levels at two-hours post-infection for the nt10100 mutant are ~2-fold less than that of wild type SINV; however, the nt10400 mutant has surpassed that observed for wild type SINV by approximately 0.7-fold. To ensure that the mutations had not compromised the inherent translatability of the genomic RNAs we checked translation using an *in vitro* system (Supporting Information, S2 Fig). No differences in translation from the mutant and WT genomic RNAs were observed *in vitro*.

Together, these data support the conclusion that the C:R interaction sites are important to early genomic RNA function during viral infection. Indeed, mutation of the individual C:R interaction sites reduces the RNA stability of the incoming genomic RNA; and, at least for nt10100 and nt10400, reduces or delays the translational activity early during infection. However, the deficit created by the mutation of the C:R interaction sites appears to be specific to the early stages of the viral molecular lifecycle. This notion is supported by the observation that, at late stages of infection, the steady state levels of the three viral RNA species are unperturbed, and the viral gene expression profiles are more or less equivalent. Therefore, we posit that the C:R interactions are critical for molecular events at the earliest stages of viral infection, and that the C:R interaction sites represent a means by which the incoming viral genomic RNA is stabilized, and perhaps licensed for translation. Once this initial critical event is surpassed, the biological role of the C:R interaction sites become less essential for reasons currently unclear. Regardless, diminished viral genomic RNA function early during the viral lifecycle appears to significantly impact the progression of infection [19, 34].

### Capsid:RNA interaction mutants induce a strong type-I IFN response

**Fig 6:**
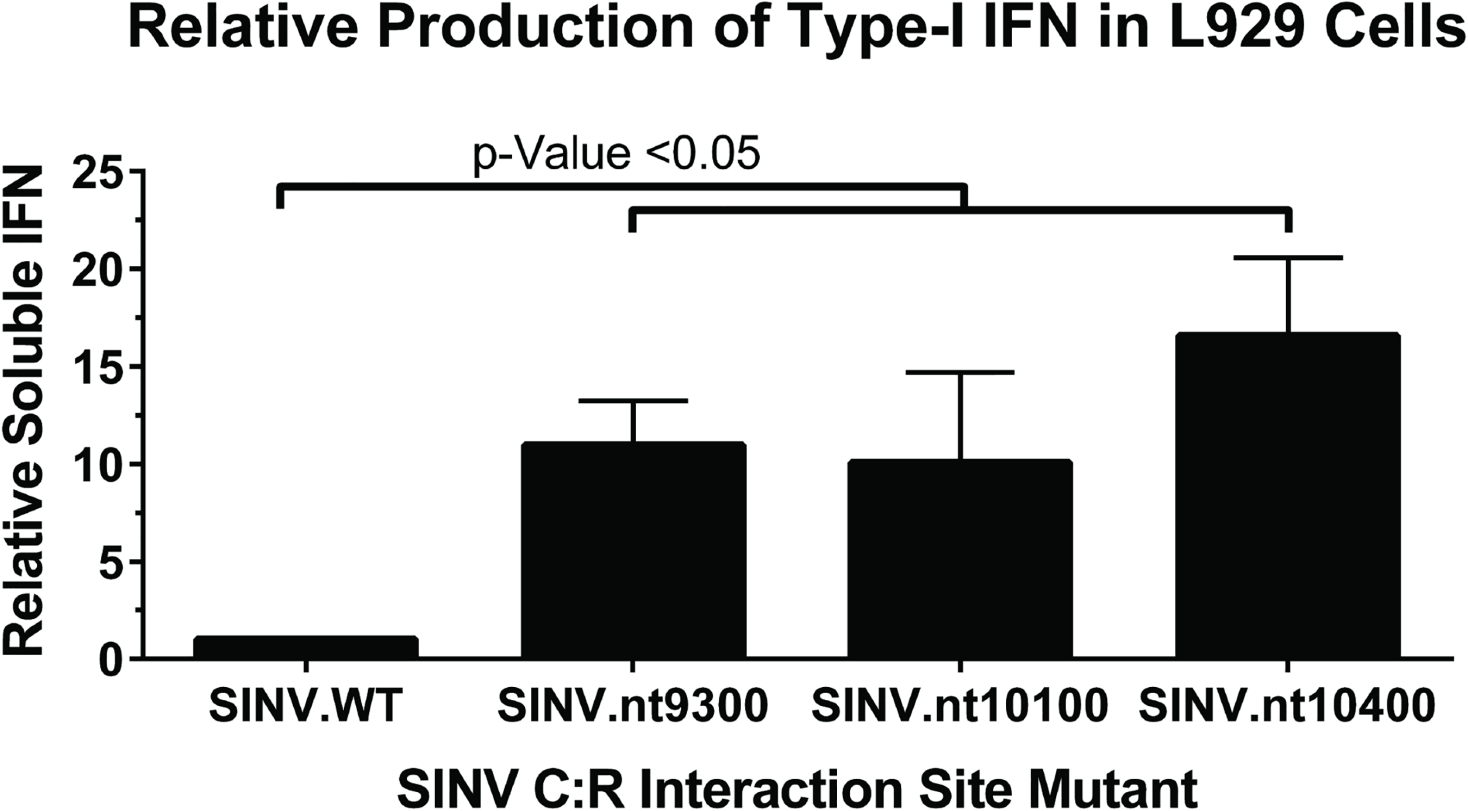
Mutation of the individual SINV C:R interaction sites increases the induction of type-I interferons. The amount of soluble Type-I Interferons produced during individual SINV C:R interaction site mutant relative to that of parental wild type virus at 24 hours post infection as determined using a tissue culture model of IFN induction. All quantitative data in this figure represents the mean of three independent biological replicates, with the error bar representing the standard deviation of the mean. Statistical significance, as indicated on the individual panels above, are the p-Values obtained from Student’s t-test.

Previously, we demonstrated that the rapid RNA decay of alphaviral genomic RNAs correlated with an increased elicitation of a type-I IFN response [34]. To determine if the SINV C:R interaction site mutants induced a more robust IFN response we quantified the soluble type-I IFN produced during infection using a tissue culture model [34, 36, 37]. As reported in Fig 6A, the individual C:R interaction site mutants produced on average 10-fold more soluble type-I IFN relative to wildtype SINV infection.

The induction of a robust IFN response is undoubtedly a significant consequence of the mutation of the cytoplasmic C:R interaction sites. Indeed, the instability of the viral RNA and apparent reduction of translational capacity early during infection likely contributes to the inability of the virus to successfully limit the induction of a soluble type-I IFN response. Together, these findings indicate that the C:R interaction site mutants are liable to be highly restricted in IFN competent systems [36, 38–43].

### Neurotropic Sindbis virus is significantly attenuated in a mouse model

**Fig 7:**
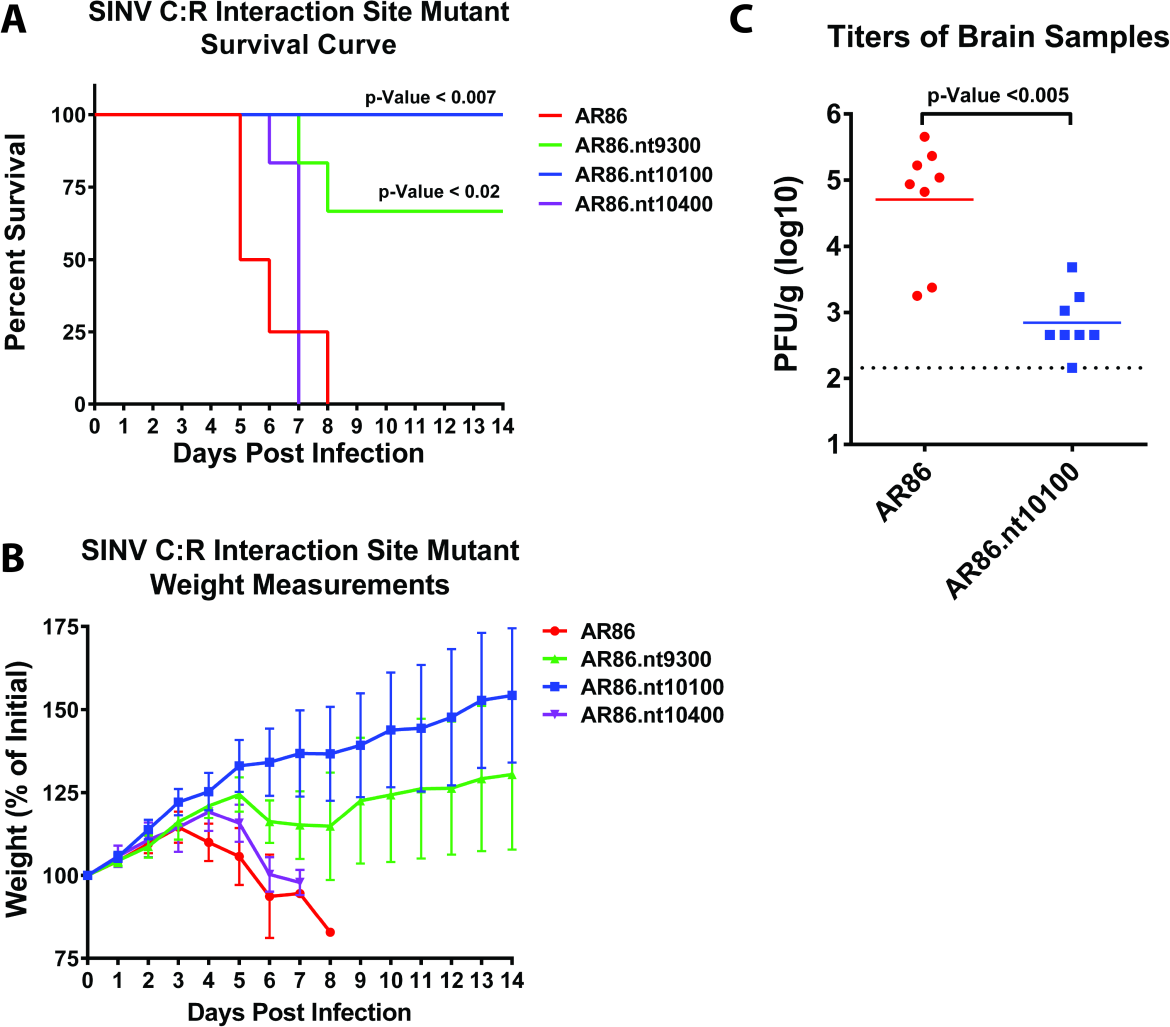
Mutation of the individual SINV C:R interaction site mutants alters SINV disease and mortality in murine models of infection. **A**) Adult wild type mice were infected with either parental SINV AR86 (N = 4), or one of the individual SINV C:R interaction site mutants, SINV.nt9300 (N = 6); SINV.nt10100 (N = 4); SINV.nt10400 (N = 6), via footpad injection. The infected animals were monitored daily for weight and disease presentation. Plotted in this panel is the percent survival for the experimentally infected animals with respect to time. Statistical analysis, via Kaplan-Meier, is reported inset to the panel. **B**) The mean weight, as plotted as percent of starting weight, with respect to time for the animals described in panel A. Plotted values represents the means of the individual animals and the error bar represents the standard deviation of the means. Animals lost to mortality were culled from further measurements. **C**) The titer of central nervous tissue of parental wild type SINV or the highly attenuated SINV.nt10100 mutant infected mice. Plotted are the individual titers of 8 experimentally infected mice harvested at 3-days post infection. Statistical significance, as indicated on the individual panels above, are the p-Values obtained from Student’s t-test.

The IFN findings described above suggested that C:R interaction site mutants would be attenuated, at least in regards to replication, in IFN competent models of infection, including immunocompetent WT mice. To test this hypothesis, we characterized the SINV C:R interaction site mutant viruses in a mouse model of infection. As shown in Fig 7, WT C57BL/6 mice infected with parental wild type AR86 SINV, which uniquely among SINV strains remains neurovirulent in adult WT mice [44], displayed significant mortality and weight loss, with a median time to death of ~5.5 days post infection (Fig 7A and 7B). In contrast, the nt9300 C:R interaction site mutant virus was significantly attenuated relative to wild type infected mice, with only a fraction of the animals infected with nt9300 mutant virus succumbing to disease. Analysis of SINV.nt9300 infected mice indicated that the disease associated with infection was mild compared with wild type AR86-infected mice as indicated by the timing and severity of weight loss. Similarly, but to a much greater extent, the SINV.nt10100 mutant virus was also attenuated in wild type mice as none of the SINV.nt10100 infected animals succumbed to infection, or demonstrated overt signs of disease as indicated by the absence of weight loss. It is important to note that these animals were productively infected with SINV, as infectious units were recovered from central nervous system tissue. As shown in Fig 7C, the viral load detected in the brains of SINV.nt10100 infected mice was decreased greater than 100-fold relative to wild type AR86 infected mice.

In contrast to SINV.nt10100 and SINV.nt9300, mice experimentally infected with SINV.nt10400 exhibited mortality comparable to wild type AR86-infected animals; however, disease progression was delayed with a median time to death of ~7 days post infection (Fig 7A). Indeed, as indicated by animal weight loss and health monitoring, the disease progression of SINV.nt10400 was distinct from that observed in mice infected with wild type AR86 as SINV.nt10400 infected animals exhibited delayed weight loss and experienced severe rapid onset paralysis at day 7, requiring the animals to be humanely euthanized (Fig 7B).

Collectively, these data suggest that the SINV C:R interaction sites identified via CLIP-seq are biologically important to infection in tissue culture models of infection and *in vivo*. Given the behavior of the SINV C:R interaction site mutants in tissue culture cells, diminished viral replication in an *in vivo* model was anticipated. The obvious difference in disease phenotype for the 9300 and 10100 C:R mutants versus the 10400 C:R mutant indicates there is an unappreciated degree of complexity in the regulation of viral processes associated with the C:R interaction.

## Discussion

The alphaviral capsid protein is a ~26kDa protein which surrounds the viral genomic RNA in viral particles and in the cytoplasm in the form of nucleocytoplasmic cores. Architecturally, the alphaviral capsid protein is globular in nature with an N-terminal domain that is implicated in RNA binding and dimerization [24, 26]. The alphavirus capsid protein is expressed as part of the structural polyprotein, which due to the serine-like protease activity of the C-terminal domain of the capsid protein, is autoproteolytically processed into free capsid protein [10]. In addition to its well-known roles in particle assembly, the alphavirus capsid of new world alphaviruses, in particular Venezuelan Equine Encephalitis virus (VEE), is also directly involved in the shutoff of host macromolecular synthesis via the interruption of nuclear export [45, 46]. Nonetheless, a larger role for the alphavirus capsid protein in the regulation of alphaviral infection has not been previously described; and, hence, the observations described in this report represent a novel contribution of the C:R interaction during alphaviral infection and pathogenesis.

### Positive-sense RNA virus capsid proteins: More than just packaging

In many positive-sense RNA viruses the viral capsid proteins serve additional roles for the viral capsid protein / RNA interactions outside of the context of particle assembly. These functions include the regulation of viral translation and RNA synthesis. For instance, the MS2 Coat Protein dimer binds to the viral genomic RNA of the MS2 bacteriophage to regulate the expression of the viral RNA-dependent RNA polymerase [47–49]. Similarly, the core protein of Hepatitis C virus (HCV) binds to the 5’ IRES during infection where it modulates the level of translation in a seemingly concentration dependent context [50–54]. Furthermore, a similar mechanism of translational regulation has been reported for other members of the alphavirus-like superfamily, in particular members of the Bromoviridae, including Brome Mosaic virus (BMV) [55, 56]. However, unlike these previous reports where the capsid protein expressed during the course of infection serves to regulate viral gene expression, the observations reported here indicate that the incoming capsid protein’s association with the SINV genomic RNA is necessary for viral RNA translation early during infection. Therefore, this phenomenon appears to be more similar to that previously described for Alfalfa Mosaic virus (AMV), where the association of the AMV capsid protein with distinct elements of the 3’UTR is required for RNA function [57–59]. Nonetheless, in contrast to AMV, the regulatory binding sites for SINV are found within the structural coding region of the viral RNAs. It is worth mentioning that this ORF, in the context of the genomic RNA, is indeed part of a large non-translated region.

Examples of capsid protein / RNA interactions that regulate RNA synthesis can readily be found in BMV, AMV, and with members of the Coronaviridae. Binding of the capsid proteins of AMV and BMV to RNA regulatory elements have been implicated in the regulation of viral RNA synthesis and promoter recognition [55, 60]. Additionally, members of the Coronaviridae require functional N protein, likely via the association of the highly charged N-terminal domain with the transcription-regulating sequences, to achieve efficient viral replication [61–64]. Nonetheless, from the data presented here we are unable to assign a role for an alphaviral C:R interaction in regards to viral RNA synthesis. For, as reported above, the accumulations of the viral RNA species is similar between the individual C:R interaction mutants and wild type parental virus. This implies that RNA synthesis / promoter utilization is not negatively affected. However, it remains possible that degeneracy between the individual C:R interaction sites is able to compensate for this activity. Unfortunately, the development of combined C:R mutants has, so far, been unsuccessful.

Several studies have described interactions between the alphaviral capsid protein and host ribosomal RNAs. Indeed, co-sedimentation studies indicated that the host 60S rRNAs interact with viral capsid proteins in vertebrate cells during infection [65, 66]. This interaction has been proposed to be a leading mechanism in particle disassembly [66]. Nonetheless, these interactions have not been exhaustively characterized and further examination is needed to complete the mechanistic understanding of alphaviral nucleocapsid disassembly.

### Alphavirus capsid / RNA interactions: A key regulator of viral RNA function

Collectively, the observations reported here are indicative of a novel role for the C:R interactions in regard to the function of the SINV genomic RNA early during viral infection. As demonstrated by the data in this report, mutation of the individual candidate C:R interaction sites identified via CLIP-seq screen significantly reduced the function of the genomic RNA early during infection. A key novel observation of these studies is that the mutation of the C:R interaction sites reduced the RNA stability of the incoming viral genomic RNA. A role for a viral capsid protein in the stabilization of viral RNAs, to our knowledge, has not yet been described for positive sense RNA viruses.

**Fig 8:**
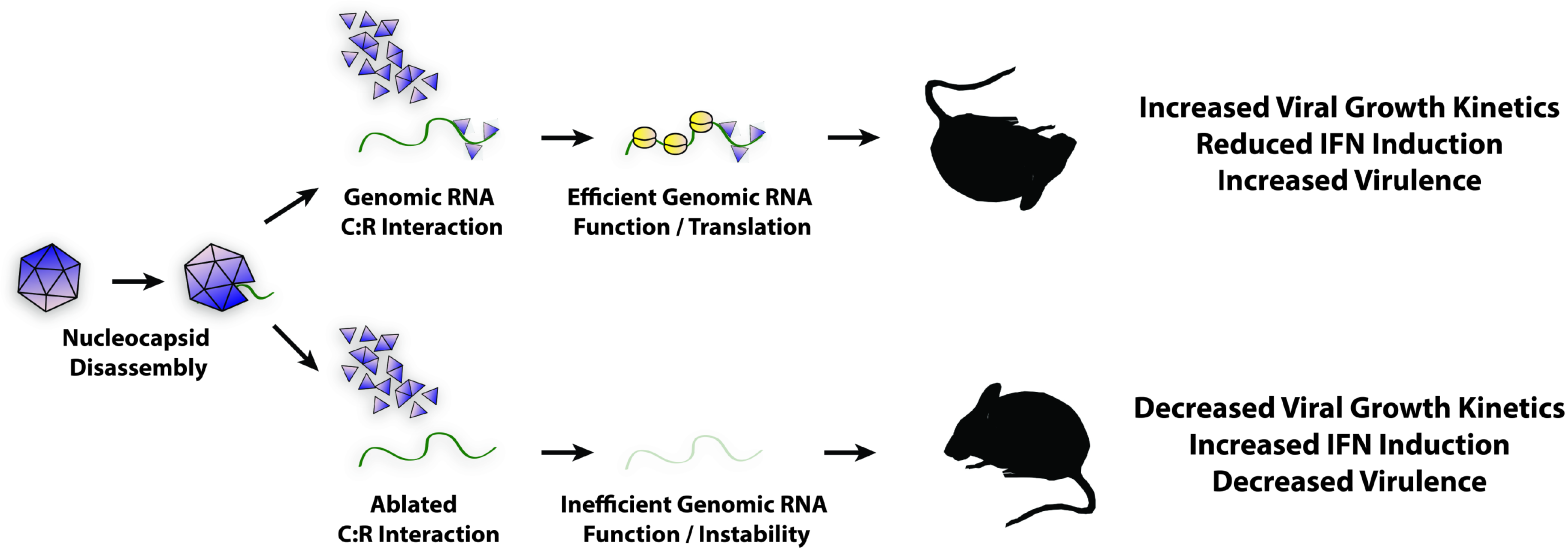
Proposed model of SINV C:R interaction site function. After receptor mediated endocytosis the acidification of the endosome resulting in release of the nucleocapsid core into the host cytoplasm. Disassembly of the nucleocapsid core occurs shortly after endosomal release; and SINV capsid protein remains bound to the C:R interaction sites following disassembly. Retention of Capsid:RNA binding at the C:R interaction sites enables evasion of the host RNA decay machinery and rapid assembly of the host translational machinery. Collectively, these interactions lead to the efficient establishment of viral infection, including but not limited to the modulation of the host innate immune response resulting in viral disease and pathogenesis. Disruption of the C:R interaction sites (lower pathway), leading to the ablation of Capsid:RNA interactions, results in RNA instability and a reduction of genomic RNA function early during infection.

The data above leads to the conclusion that the interaction sites of the SINV capsid protein with the viral genomic RNA identified in this study by CLIP-Seq, while not required for RNA function late during infection, are vital to early genomic RNA stability, and function. Moreover, mutation of the C:R interaction site diminished translation of the viral genomic RNA early during infection, likely resulting in increased type-I IFN production. Currently the precise mechanism of C:R-mediated regulation is unclear; however, the C:R interaction sites are clearly involved in the stabilization of the incoming viral genomic RNA and the regulation of early viral translation. We suggest a model in which capsid protein of incoming nucleocapsid complexes remains associated or re-associates with specific sites in the genome following nucleocapsid disassembly enhancing RNA stability and facilitating translation (Fig 8). This is a working model, whether the effects on RNA function are due only to capsid binding, or could be affected by the binding of other RNA binding proteins at proximal or overlapping sites is unknown. Ongoing studies in the Sokoloski and Hardy laboratories have indicated that the C:R interaction sites may be common sites of virus and host protein binding. The precise role(s) and contributions of these interactions are currently being characterized. Further potential mechanisms include direct or indirect roles in the recruitment of the translational machinery, or beneficial host RNA-binding proteins to the viral RNA; or perhaps the evasion of toll-like receptors and RNA-helicase sensors such as RIG-I and MDA5, or the eluding of the host cellular RNA surveillance machinery such as the host nonsense-mediated RNA decay pathway. While our data cannot completely rule out this possibility, we do not believe that the decreased RNA stability observed in the C:R interaction site mutants is due to nonspecific recruitment of cellular endonucleases (for instance RNAse L), as mutation of off-target sites does not result in an altered phenotype (Supplementary Information, S3 Fig). Whereas, the mutation of the C:R interaction sites reduced viral growth kinetics, likely as a result of decreased infectivity due to impaired genomic RNA function early during infection. In addition to their molecular role(s), the C:R interaction sites represent a set of previously unidentified pathogenicity determinants in that mutation of the C:R interaction sites significantly attenuates viral infection *in vitro* and *in vivo*. As shown in this study mutation of the SINV C:R nt9300 and nt10100 interaction sites reduced the viral load, pathology, and morbidity of a neurotropic SINV infection. These findings have great potential as a means by which viruses may be attenuated for the purpose of rational vaccine development. Given that C:R interaction site mutation limits viral RNA function early during infection, but not late during infection, implies that viral dissemination but not immunogenicity would be limited. However, further exploration is necessary, as not all C:R interaction sites resulted in complete attenuation; as the SINV.nt10400 mutant exhibited significant mortality and morbidity in experimentally infected mice. Currently, the precise mechanism behind this phenotype is unclear. One possibility being investigated is that this site is also bound by a host factor and this contributes to the phenotype observed with this particular mutant. It should be noted that while the C:R interaction at 10400 obviously is important in maintaining genome stability following infection the site may also play a role in the function of the viral subgenomic mRNA that this may be affected by the binding of a host factor.

### Alphavirus capsid / RNA interactions: Potential insights to alphaviral assembly?

Several studies have indicated that the association of the alphaviral capsid protein with viral RNA is nonspecific in regards to primary nucleotide sequence and secondary structure, and is likely driven by electrostatic interactions between the N-terminus of Capsid and the phosphodiester RNA backbone [24–28]. While these studies primarily focused on the interactions of nucleic acids and the viral capsid proteins *in vitro*, it is highly likely that these observations are valid during *bona fide* infections as evidenced by the distributive nature of the Capsid:RNA interactions observed in particles. However, collectively the data presented in this report indicate that the interactions, or at least the strength thereof, between the SINV capsid protein and the genomic RNA may be context dependent. The disseminated pattern of binding of the viral genomic RNA to capsid observed in purified viral particles supports a model where the C:R interactions are largely nonspecific, and perhaps based on charge-charge interactions [25, 27, 28]. However, from this data set it is impossible to identify if the interactions between the capsid protein and cargo RNA are mutually saturating (where each capsid monomer interacts with ~50 nt of genomic RNA), or unsaturated (where not every nt is associated with capsid monomer) but nonspecifically distributed along the length of the genome. Further examination of the pattern of binding observed in purified viral particles reveals an interesting phenomenon- there are two regions of the SINV genome where sequence coverage is greatly decreased. These regions roughly correspond to the previously described Packaging Signal, and the subgenomic promoter region [18, 67, 68]. Importantly, the decreased coverage of these regions cannot be simply explained by bias in the library generation (as is the case for the extreme 5’ and 3’ termini of the genome). In these instances, the decrease in coverage was due to genomic RNA having a 5’ cap and 3’ poly(A) tail, which effectively prevented the linkage of the 5’ and 3’ adaptors as these RNA features were not modified prior to library construction. Nonetheless, decreased sequence coverage at the Packaging Signal could be potentially explained due to the presence of a high degree of secondary structure within the element. The nuclease fragmentation approach utilized in these studies is selective for single-stranded RNA, therefore regions with a high degree of dsRNA may be excluded from nuclease digestion. The net result would be the formation of RNA fragments that lay outside the region of interest / selection during the preparation of the cDNA libraries. Alternatively, if the capsid interaction is exceptionally avid at this location the fragmentation of the viral RNA may be similarly diminished, resulting in decreased library coverage at the Packaging Signal. Further analysis of the decreased coverage at the cis-acting sites is separate and ongoing research focus.

Examination of the interactions between the capsid protein and the viral genomic RNA in a cytoplasmic context indicated that several discrete interaction sites were readily observable. However, from our data we cannot conclude as to the molecular context of these interactions, as the milieu of capsid interactions likely expands from monomers (or as some models suggest dimers) to complete intact nucleocytoplasmic cores. From our current data it is impossible to determine the molecular nature of the interaction in regards to protein:RNA stoichiometry and molecular context. Unfortunately, given the promiscuous interaction of alphaviral capsid proteins with nucleic acids (including DNA) deciphering the supramolecular nature of the interaction through a reductionist in vitro approach presents significant challenges [25, 27, 28]. Moreover, it is unclear if these interactions are exclusive of other C:R binding events, or simply the cytoplasmic C:R interactions with high relative affinities. It should be noted that the method of cross-linking used in these studies is inefficient, which increases the specificity but reduces overall signal intensity during the CLIP-seq analyses; it is therefore likely that the observed cytoplasmic C:R interaction sites are exceptionally stable relative to other C:R interactions.

## Materials and methods

### Tissue culture cells

BHK-21 (gift from Charles Rice, Rockefeller University), HEK293, and L929 cells (both cell lines were gifts from Pranav Danthi, Indiana University) were cultured in Minimal Essential Media (MEM, Cellgro) supplemented with 10% Fetal Bovine Serum (FBS, Atlanta Biologicals), 1x antibiotic / antimycotic solution (Cellgro), 1x nonessential amino acids (Cellgro) and L-glutamine (Cellgro). All cell lines utilized in this study were cultured at 37° in the presence of 5% CO_2_.

### Preparation and purification of SINV virions

Wild type SINV Toto1101, SINV p389 (a Toto1101 derived strain containing GFP in frame with nsP3), SINV pToto1101-naluc (a Toto1101 derived strain containing nanoluciferase in frame with nsP3), wild type SINV AR86, and all derivatives thereof (described below), were prepared by electroporation as previously described [34]. Briefly, 10 ug of *in vitro* transcribed RNA were electroporated into BHK-21 cells via a single pulse from a Gene Pulser Xcell system (Bio-Rad) at 1.5kV, 25mA and 200Ohms. After the development of cytopathic effect (typically 24 to 36-hours post-electroporation) the tissue culture supernatants were collected and clarified via centrifugation at 8,000xg for 10 minutes at 4°C. The resulting stocks were aliquoted and either used immediately or stored at -80°C for later use.

SINV particles were purified via a two-step process [19]. First, supernatants from 2x10^8^ HEK293 cells infected with SINV Toto1101 at an MOI of 3 PFU/cell were harvested at 18-hours post infection. The viral particles were concentrated via pelleting through a 27% (m/v) sucrose cushion prepared in HNE buffer (20mM HEPES [pH 7.4] / 150mM NaCl / 5mM EDTA) by centrifugation at 185,000xg for 1.5 hours in a 60Ti rotor. Second, the pelleted viral particles were then resuspended in HNE buffer and applied to a 30% / 60% (m/v) sucrose step gradient prepared in HNE. The SINV particles were then banded via centrifugation at 250,000xg for 2.5 hours in an SW41 rotor at 4°C. The purified SINV particles were collected via needle aspiration, aliquoted and stored at -80°C for later use.

### Cross-linked immunoprecipitation–deep sequencing (CLIP-seq) of SINV C:R complexes

Per CLIP-seq library, either purified SINV particles or infected tissue culture cells were cross-linked via exposure to shortwave (254nm) UV irradiation. Briefly, approximately 10^11^ SINV Toto1101 particles in a volume of 500μl, or 2x10^7^ HEK293 cells infected with SINV Toto1101 at an MOI of 5 PFU/cell at 18hpi, were irradiated with 5700x100 μjoules per square centimeter in a Stratalinker. After cross-linking the RNA-protein complexes were solubilized in RIPA buffer (50mM Tris [pH 7.6] / 150mM NaCl / 1.0% NP-40 / 0.5% Sodium Deoxycholate / 0.1% SDS) and frozen at -80°C for later use. Prior to immunoprecipitation the lysates were thawed on ice, vortexed and clarified via centrifugation at 16,000xg for 5 minutes at 4°C. The clarified supernatants (~500μl) were transferred to a fresh tube. The cross-linked lysates were then incubated with 50ul packed volume of Protein G sepharose beads for 15 minutes at 4°C prior to being clarified via centrifugation at 5,000xg for five minutes. The resulting pre-blocked lysates were then immunoprecipitated using a polyclonal anti-capsid antibody, or control rabbit IgG sera. The cross-linked RNA:protein complexes were incubated in the presence of antibody at 4°C for a period of 2 hours under constant agitation. After antibody binding, 100ul packed volume of Protein G sepharose was added to each sample and further incubated for another 2 hours. After resin binding the RNAs were fragmented via the addition of RNAse T1 (ThermoScientific) to each immunoprecipitation. The fragmentation reactions were allowed to incubate for 15 minutes at room temperature. After fragmentation, the immunoprecipitated complexes were purified via centrifugation at 5,000xg for 2 minutes and washed three times with RIPA buffer and twice more with sterile 1xPBS. The resulting purified, fragmented RNA:protein complexes were treated with Proteinase K for 30 minutes at 37°C to release the RNA fragments from the immunoprecipitated complexes. The purified RNA fragments were then extracted using TRIzol and resuspended in a minimal volume. The RNA fragments were then used as the input materials for cDNA library generation via the NEBNext sequencing kit, as according to the manufacturer’s instructions. The resulting cDNA libraries were sequenced using the MiSeq platform.

The specificity of the immunoprecipitation protocol described above was confirmed independently using small scale analytical replicates and metabolic labeling. HEK293 cells were either mock treated, or infected with SINV at an MOI of 10 PFU/cell, and allowed to incubate for 16 hours under normal conditions. Thirty minutes prior to the labeling period the infected monolayers were washed twice with 1xPBS and the media was replaced with methionine / cysteine free DMEM supplemented with 5% dialyzed FBS. After the depletion period the media was removed and replaced with methionine/cysteine free DMEM supplemented with L-AzidohomoAlanine (L-AHA) at a concentration of 50um. The cells were allowed to incubate under normal conditions for a period of two hours prior to removal of the culture media. Prior to harvesting the monolayers were washed three times with 1xPBS. Whole cell lysates were generated via the addition of RIPA buffer. Protein concentration was quantified via Bradford Assay and equivalent amounts of protein were precipitated via methanol / chloroform treatment. The precipitated proteins were resuspended in 1xPBS containing 5uM DIBO-AlexaFluor 64 and incubated for 1 hour at room temperature while shielded from light.

After the labeling period, the samples were diluted in 2x volumes of RIPA buffer and immunoprecipitated identically to that described for the CLIP-Seq experiments. These treatments only differed on the basis of scale- as the total materials used were reduced 100-fold relative to those used for library generation. After immunoprecipitation 6x Laemmli buffer was added to a final concentration of 2x and the samples analyzed via SDS-PAGE. Protein species were detected via a Pharos Molecular Imager using the appropriate excitation and emission detection settings. As shown in Supporting Information (S1 Fig), the anti-capsid sera specifically immunoprecipitated the SINV capsid protein from infected extracts. In addition, no other host proteins were enriched during immunoprecipitation with anti-capsid sera in either infected or mock infected lysates. Furthermore, the nonspecific antibody control did not enrich for any host, or viral, protein. Collectively these data are indicative of the specificity and purity of the immunoprecipitations used for cDNA library generation.

The cDNA libraries were trimmed to remove indices and adaptors prior to alignment to a reference genome consisting of the parental Toto1101 strain of SINV. Alignments were performed using LAstZ with standard parameters. Only reads corresponding to the positive-sense genomic RNA were mapped to the reference genome sequence. Sequence coverage was clustered at the nucleotide level of resolution from the aligned sequence reads. Analysis of the clustered sequence data relied on a subtractive method whereby the sequence coverage of capsid-specific libraries was compared to nonspecific control libraries. For these analyses the coverage of both the Capsid and Nonspecific control libraries were normalized internally by dividing each base by the total number of represented nucleotides to gain a percentage which was then multiplied by an arbitrary amount to generate a standardized measure of each nucleotides relative representation amongst independent library sets. The resulting values from the control libraries were averaged and subtracted from the Capsid-specific libraries to generate a difference map (as shown in Fig 1C). Z-scores for each individual nucleotide were calculated from the statistical mean and standard deviation from the subtractive analysis data sets. For these studies statistical significance was rigorously defined as nucleotide clusters with differential coverage values greater than 5 standard deviations from the mean, which correlates to a Z-score of ~10^−25^. The sequencing data directly relevant to the studies described herein are available accompanying this document as S1 Data. This supplemental information also includes a statistical summary of the entire RNA sequencing dataset and analysis of read size for the regions of interest and a control region. The entire RNA sequencing dataset has been deposited in the National Center for Biotechnology Information (NCBI) Gene Expression Omnibus, and can be accessed using the following URL: https://urldefense.proofpoint.com/v2/url?u=http-3A__www.ncbi.nlm.nih.gov_geo_query_acc.cgi-3Facc-3DGSE99879&d=AwIEAg&c=SgMrq23dbjbGX6e0ZsSHgEZX6A4IAf1SO3AJ2bNrHlk&r=OEUL-Bhg_4jju0S0FtlcvxycNjpESQ60pJohUAWMntY&m=S87220zYVRm6qbr6ME7XEYiR1DQZkoyOpa4rz4m8xmQ&s=M1W8fvnSVBtk9BsSVmlJIUA0pXouRfnBZuKIY4xy5jg&e=

### Development of SINV C:R interaction site mutants

Following the identification and prioritization of the nt9300, nt10100, and nt10400 C:R interaction sites mutant SINV strains were developed using the Q5 site directed mutagenesis kit (NEB). Briefly, PCR amplification of the parental Toto1101, or AR86, infectious cDNA clones were performed using the sequences indicated in Fig 2. Q5 reaction products were confirmed visually using standard agarose electrophoresis and diagnostically digested to confirm product size. The individual C:R interaction site mutant SINVs were completely sequenced to confirm the presence of the mutation and sequence relative to wild type virus prior to being utilized as templates for the production of infectious virus as described above.

### Quantitative immunoprecipitation

The quantitative assessment of the capsid:RNA interactions of the individual C:R interaction sites for parental and C:R interaction site mutants utilized small scale extracts generated identically to that described above for the CLIP-seq process. Briefly, HEK293 cells were infected at an MOI of 5 prior to cross-linking via UV irradiation. After the preparation of whole cell lysates, the capsid:RNA complexes were immunoprecipitated and the bound RNAs fragmented with RNAse T1. The immunoprecipitates were washed extensively and the retained RNA fragments were release via proteinase K digestion. The RNA fragments were extracted via TRIzol according to the manufacturer’s instructions.

The precipitated RNAs and paired input controls, were used as the input materials for reverse transcription using random hexamer. The resulting cDNAs were assessed quantitatively via qRT-PCR as described below using the following primer pairs:

SINV.nt9300F 5’-GCACCGCCATCAAGCAATGTGTGGC-3’; SINV.nt9300R 5’-CAATTTCCCTTGGGCCGTGTGGTCG-3’;
SINV.nt10100F 5’-TGTTCCAAATGTGCCACAGATACCG-3’; SINV.nt10100R 5’-AATGTACTCTTGGTTGGTGGAAGGC-3’;
SINV.nt10400F 5’-CAGCAGATTGCGCGTCTGACCATGC-3’; SINV.nt10400R 5’-GACTCCGTTCACGTACACATCTAGG-3’.

### One-step growth kinetics

The viral growth kinetic assays performed in these studies were essentially as previously described [34]. Briefly, HEK293 cells were infected with either wildtype or C:R interaction site mutant viruses at a multiplicity of infection of 10 PFU/cell. The infected monolayers were washed with 1xPBS to remove unbound virus and fresh whole media was added. At regular times post infection the tissue culture supernatant was harvested and replaced with fresh growth media. All samples were frozen at -80°C until completion of the time course. Viral titers were determined using the standard plaque assay protocols involving BHK-21 cells and a 1% agarose overlay. Plaque assays were allowed to incubate until plaques were readily visible prior to being fixed with 3.7% formaldehyde and stained with crystal violet.

### Viral RNA quantification / determination of particle number

At the indicated times post infection, infected HEK293 cells were washed twice with 1xPBS and harvested into TRIzol. Total RNA was extracted according to the manufacturer’s directions, with carrier glycogen being added to the precipitations. Equal amounts of total RNA (0.5μg) were used as template for the synthesis of cDNA and assessed quantitatively via qRT-PCR using the method and oligonucleotide primers previously described [34, 69, 70]. Briefly, the positive and negative sense viral RNAs were selectively reverse transcribed using specific RT primers. The absolute quantities of the genomic and total positive sense viral RNAs (consisting of the genomic and subgenomic RNAs) were determined using paired standard curves; and the absolute quantity of the subgenomic RNA itself was determined via subtraction of the number of genomes from the total positive sense RNAs. The minus strand was detected in isolation from the positive sense RNAs. All qRT-PCR reactions were normalized to the level of 18S rRNA present as previously described [34, 69, 70].

### Viral infectivity assays

To determine the relative infectivity of each SINV C:R interaction site mutant the number of total viral particles per unit volume, as measured by the genome equivalents per ml, was determined using qRT-PCR, as previously described [19]. Briefly, the viral genomic RNAs from BHK-21 tissue culture cell supernatants were utilized as the source template for cDNA synthesis. The resulting cDNAs were then assessed using qRT-PCR and the absolute quantity of viral particles per unit volume determined using standard curve analysis. The number of infectious units was determined using standard plaque assay on BHK-21 cells, and the infectivity was determined by comparing the total number of particles per infectious unit.

### Viral gene expression

The quantitative analyses of viral gene expression described in these studies was performed via one of two methods-

Gross viral and cellular gene expression was determined via metabolic labeling of infected cells [71, 72]. Briefly, HEK293 cells were infected with SINV at an MOI of 10 PFU/cell and allowed to incubate for the indicated times under normal conditions. Thirty minutes prior to the labeling period the infected monolayers were washed twice with 1xPBS and the media was replaced with methionine / cysteine free DMEM supplemented with 5% dialyzed FBS. After the depletion period the media was removed and replaced with methionine/cysteine free DMEM supplemented with 35S-labeled methionine and cysteine at a specific activity of 50μCi/ml. The cells were further incubated under the labeling conditions for the indicated time periods. At the end of the labeling period the cells were washed three times with 1xPBS prior to lysis in RIPA buffer. Equal volumes of cell lysates were analyzed by SDS-PAGE and radiolabeled proteins were detected using standard phosphorimaging practices.

Quantitative analysis of genomic RNA gene expression early during infection was performed using a process previously described, with one notable exception [34, 73]. Specifically, the reporter construct, and hence detection system used, consisted of an in-frame fusion of the Nanoluc reporter gene with nsP3 at a position identical to that of pToto1101-Fluc [73, 74]. Nanoluciferase signal was assayed according to the manufacturer’s instructions. All analysis methods were identical to that previously described [19].

### SINV incoming genomic RNA half-life assay

Viral RNA half-lives were assessed as previously described [34]. HEK293 cells, cultured in media supplemented with 50uM 4-thio Uridine (Sigma), were infected with either parental SINV Toto1101 or the individual SINV C:R interaction site mutant SINVs at an MOI of 5 PFU per cell. At the indicated times post infection the tissue culture supernatant was removed and the cell monolayers were washed three times with PBS prior to TRIzol (Invitrogen) extraction. A total of 1μg of total RNA was biotinylated using HPDP-Biotin (Pierce). The biotinylated RNAs were bound to Ultralink streptavidin resin (Pierce) to remove newly transcribed viral RNAs. The unbound RNAs were then phenol extracted and ethanol precipitated prior to use as a template for cDNA synthesis via reverse transcription using Random Hexamer. The resulting cDNAs were assayed via qRT-PCR to determine the relative abundances of the incoming SINV genomic RNAs normalized to the cellular 18S rRNA. RNA half-lives were calculated as reported in Dolken et al from data pertaining to the initial phase of decay [75].

### Quantification of soluble type-I IFN production

The production of soluble type-I IFN was assayed as previously described [34]. Briefly, the IFN competent L929 cell line was infected with the C:R interaction site mutant derivatives or parental wildtype SINV AR86 at an MOI of 10 PFU/cell for 1 hour at room temperature. Prior to further incubation the infected tissue culture cells were washed three times with 1xPBS and fresh media was added. The tissue culture supernatants were harvested 24hpi and clarified via centrifugation. Infectious viral particles in the supernatant were inactivated via acidification and UV irradiation. The inactivation of samples was confirmed via standard plaque assays. The inactivated supernatants were then serially diluted and tittered onto fresh L929 cells in a 96-well format. After a period of 24 hours of treatment the media was removed and the cells were challenged with a fluorescent Chikungunya mCherry reporter strain at an MOI of 10 PFU/cell. Viral gene expression was detected at 24hpi via a Typhoon 9200 phosphorimager and quantified via densitometry. After 96 hpi the cells were fixed and assayed for cell death via crystal violet staining; the formation of CPE was highly consistent with viral mCherry expression. Relative IFN production was calculated as a function of the dilution needed to attain a 50% reduction in viral gene expression.

### Mouse experiments

Four-week old WT C57BL/6J mice were obtained from the Jackson Laboratory. Mice were inoculated in the left rear footpad with virus in diluent (phosphate-buffered saline [PBS] supplemented with 1% FBS) in a volume of 10 μl. On the termination day of each experiment, mice were sedated with isoflurane and euthanized by thoracotomy, blood was collected, and mice were perfused extensively by intracardiac injection of PBS. Serum was obtained by collecting blood in serum separator tubes (BD). PBS-perfused tissues were removed by dissection, placed into PBS-1% FBS and homogenized using a MagNA Lyser (Roche). The amounts of infectious virus in tissues were quantified by standard plaque assays using BHK-21 cells.

### Ethics statement

This study was conducted in accordance with the recommendations in the Guide for the Care and Use of Laboratory Animals and the AVMA Guidelines for the Euthanasia of Animals. All animal experiments were performed with the approval of the Institutional Animal Care and Use Committee at the University of Colorado School of Medicine (Assurance Number: A3269-01) under protocol B-86514(10)1E. Experimental animals were humanely euthanized at defined endpoints by exposure to isoflurane vapors followed by thoracotomy.

### Statistical analyses

Unless otherwise noted, the quantitative data reported in this study are the means of a minimum of three independent biological replicates. Where appropriate, the statistical analysis of comparative samples was performed using variable bootstrapping, as previously described [76]. The error bars indicate the standard deviation from the mean. When indicated, the p-values associated with individual data sets are the result of the Student’s T-test for the corresponding data.

## Supporting information

S1 Table SINV genomes used for single nucleotide polymorphism analysis. (PDF)

S1 Dataset Sequence data used for CLIP-Seq analyses. Statistical summary of sequencing data. SINV-specific sequence reads. Read size analysis across the identified capsid interaction sites and a control region in the nsP4 coding sequence. QC was performed using Trimmomatic. Quality scores were averaged over a sliding window of 4 nucleotides and nucleotides with values less than 20 were removed.
(XLSX)

S1 Fig The Immunoprecipitation of SINV capsid protein is specific. **A)** Metabolically labeled mock control and infected HEK293 cells cell lysates were immunoprecipitated with either non-specific control sera or anti-capsid sera using conditions identical to those used for the preparation of the cDNA libraries used in CLIP-seq. The input lane represents 1/10^th^ of the starting material for the IP. Data shown is representative of several biological replicates. B) SINV infected HEK293 cells were either mock- or UV-crosslinked via shortwave UV irradiation at 18hpi. The cell monolayers were harvested via gentle scraping and solubilized in RIPA buffer to form whole cell lysates. The cell lysates were then precipitated with antibodies specific for either SINV Capsid, or control rabbit IgG, as indicated on the figure. All purification conditions were identical to those described for the development of the CLIP-Seq cDNA libraries utilized in this study, with the only exception being that RNA fragmentation was omitted. After purification, cDNA was generated from the immunoprecipitated materials, and the presence of the nsP1 coding region was detected via RT-PCR and agarose gel electrophoresis. Data shown is representative of three biological replicates.
(TIF)

S2 Fig SINV C:R interaction site mutants are unaffected in translational capacity in vitro. In vitro transcribed genomic RNAs for parental SINV, and each of the individual C:R interaction site mutants were assessed for translation using Rabbit Reticulocyte Extracts according to the manufacturer’s directions. The amount of translation was detected using nanoluciferase detection. Data shown is the mean of three independent biological replicates, with the error bar representing the standard deviation of the mean.
(TIF)

S3 Fig Mutation of an “off-target” interaction site does not affect viral growth kinetics. The one-step growth kinetics of parental and a non C:R interaction site mutant as observed in HEK293 cells. Briefly, a region of the SINV genomic RNA detected, but identified as statistically insignificant, by CLIP-seq analysis was mutated using identical parameters to the bona fide C:R interaction site mutants. This region corresponds to nt41-98 of the viral genomic RNA, which includes the genuine start site of nsP1. The quantitative data in this figure represents the mean of three independent biological replicates, the error bar representing the standard deviation of the mean.
(TIF)
