# Supplementary material for: Identification of Interactions between Sindbis Virus Capsid Protein and Cytoplasmic vRNA as Novel Virulence Determinants
Source: PLoS Pathog. 2017 Jun 29;13(6):e1006473. doi: 10.1371/journal.ppat.1006473 (PMC5507600; doi:10.1371/journal.ppat.1006473)
Supplement: S1 Table — (PDF) [file ppat.1006473.s001.pdf]

		GenBank	Length
Species	Strain	Accession #	(nt)
SINV	UNKNOWN-HM147984	HM147984	11739
SINV	LEIV-65A	KF981618	11592
SINV	Edsbyn 82-5	M69205	11708
SINV	UNKNOWN-J02363	J02363	11703
SINV	UNKNOWN-BD269911	BD269911	11703
SINV	UNKNOWN-CS227856	CS227856	11703
SINV	SW6562	AF429428	11569
SINV	XJ-160	AF103728	11626
SINV	ILOMANTSI-2002A	JQ771794	11554
SINV	Ilomantsi-2005M	JQ771793	11541
SINV	LEIV-9298	JQ771799	11581
SINV	JOHANNES-2002	JQ771797	11581
SINV	KIIHTELYSVAARA-2002	JQ771798	11581
SINV	ILOMANTSI-2002C	JQ771796	11581
SINV	ILOMANTSI-2002B	JQ771795	11581
SINV	28.9	GU361118	11660
SINV	5.3	GU361116	11701
SINV	Berlin-2010A	JX570540	11717
SINV	UNKNOWN-HV228506	HV228506	11740
SINV	Lovanger	KF737350	11648
SINV	hr	NC_001547 *	11703
SINV	UNKNOWN-BD269910	BD269910	11703
SINV	S.A.AR86	U38305	11663
SINV	Girdwood S.A.	U38304	11717
SINV	YN87448	AF103734	11717
