# Supplementary figures and images for: Identification of Interactions between Sindbis Virus Capsid Protein and Cytoplasmic vRNA as Novel Virulence Determinants
Source: PLoS Pathog. 2017 Jun 29;13(6):e1006473. doi: 10.1371/journal.ppat.1006473 (PMC5507600; doi:10.1371/journal.ppat.1006473)

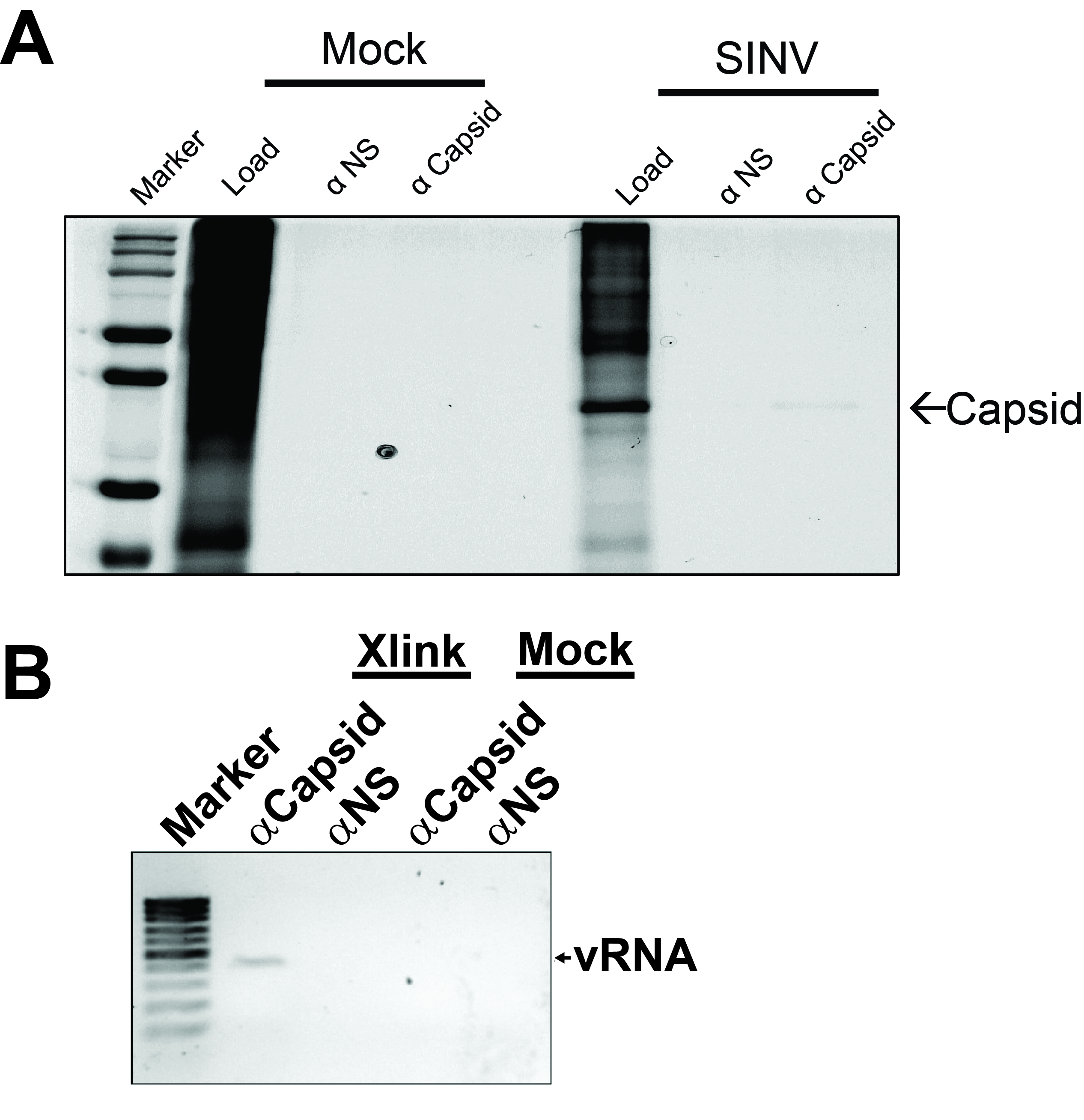

Supplement: S1 Fig — A) Metabolically labeled mock control and infected HEK293 cells cell lysates were immunoprecipitated with either non-specific control sera or anti-capsid sera using conditions identical to those used for the preparation of the cDNA libraries used in CLIP-seq. The input lane represents 1/10th of the starting material for the IP. Data shown is representative of several biological replicates. B) SINV infected HEK293 cells were either mock- or UV-crosslinked via shortwave UV irradiation at 18hpi. The cell monolayers were harvested via gentle scraping and solubilized in RIPA buffer to form whole cell lysates. The cell lysates were then precipitated with antibodies specific for either SINV Capsid, or control rabbit IgG, as indicated on the figure. All purification conditions were identical to those described for the development of the CLIP-Seq cDNA libraries utilized in this study, with the only exception being that RNA fragmentation was omitted. After purification, cDNA was generated from the immunoprecipitated materials, and the presence of the nsP1 coding region was detected via RT-PCR and agarose gel electrophoresis. Data shown is representative of three biological replicates. (TIF) [file ppat.1006473.s003.tif]

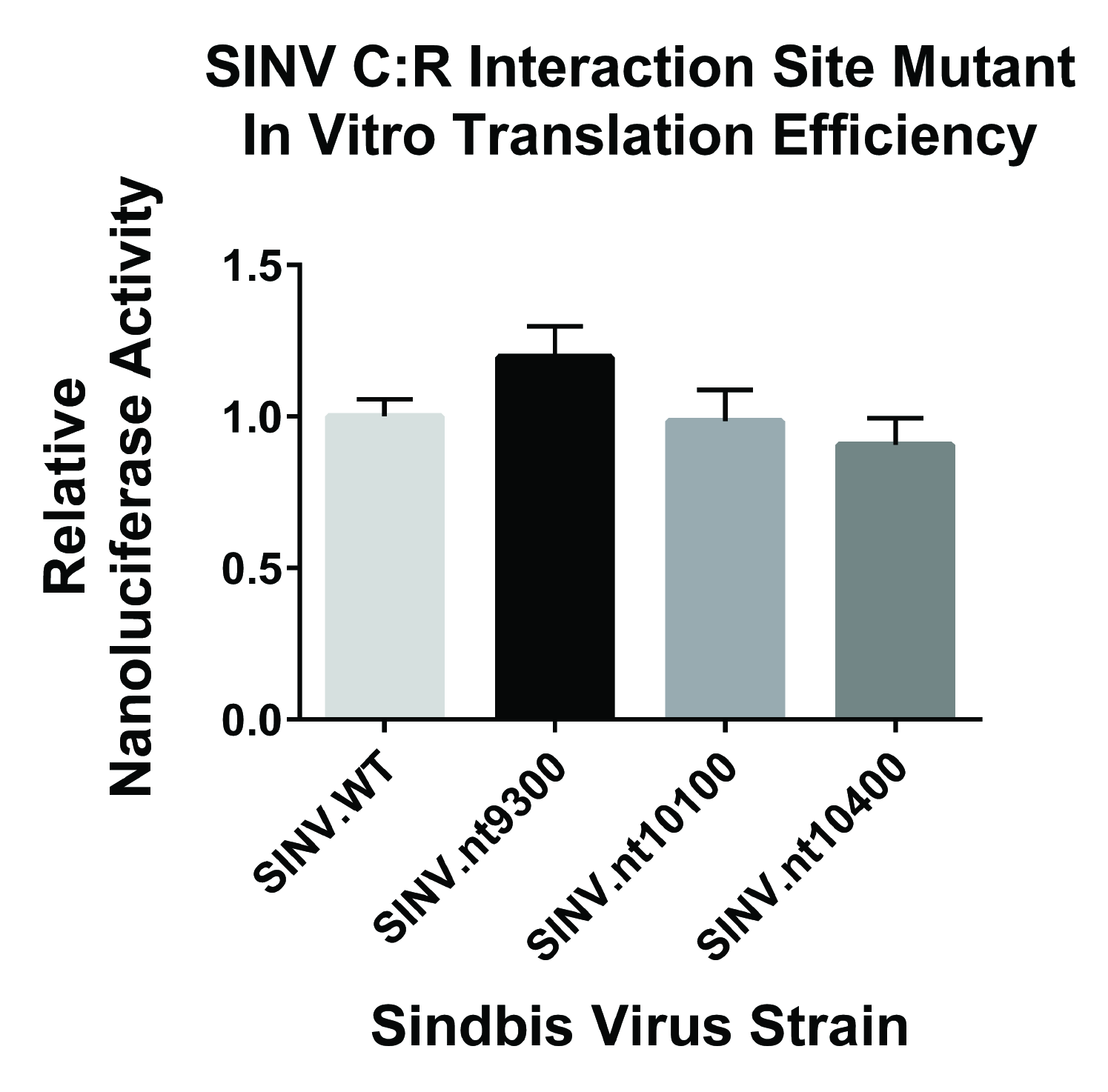

Supplement: S2 Fig — In vitro transcribed genomic RNAs for parental SINV, and each of the individual C:R interaction site mutants were assessed for translation using Rabbit Reticulocyte Extracts according to the manufacturer’s directions. The amount of translation was detected using nanoluciferase detection. Data shown is the mean of three independent biological replicates, with the error bar representing the standard deviation of the mean. (TIF) [file ppat.1006473.s004.tif]

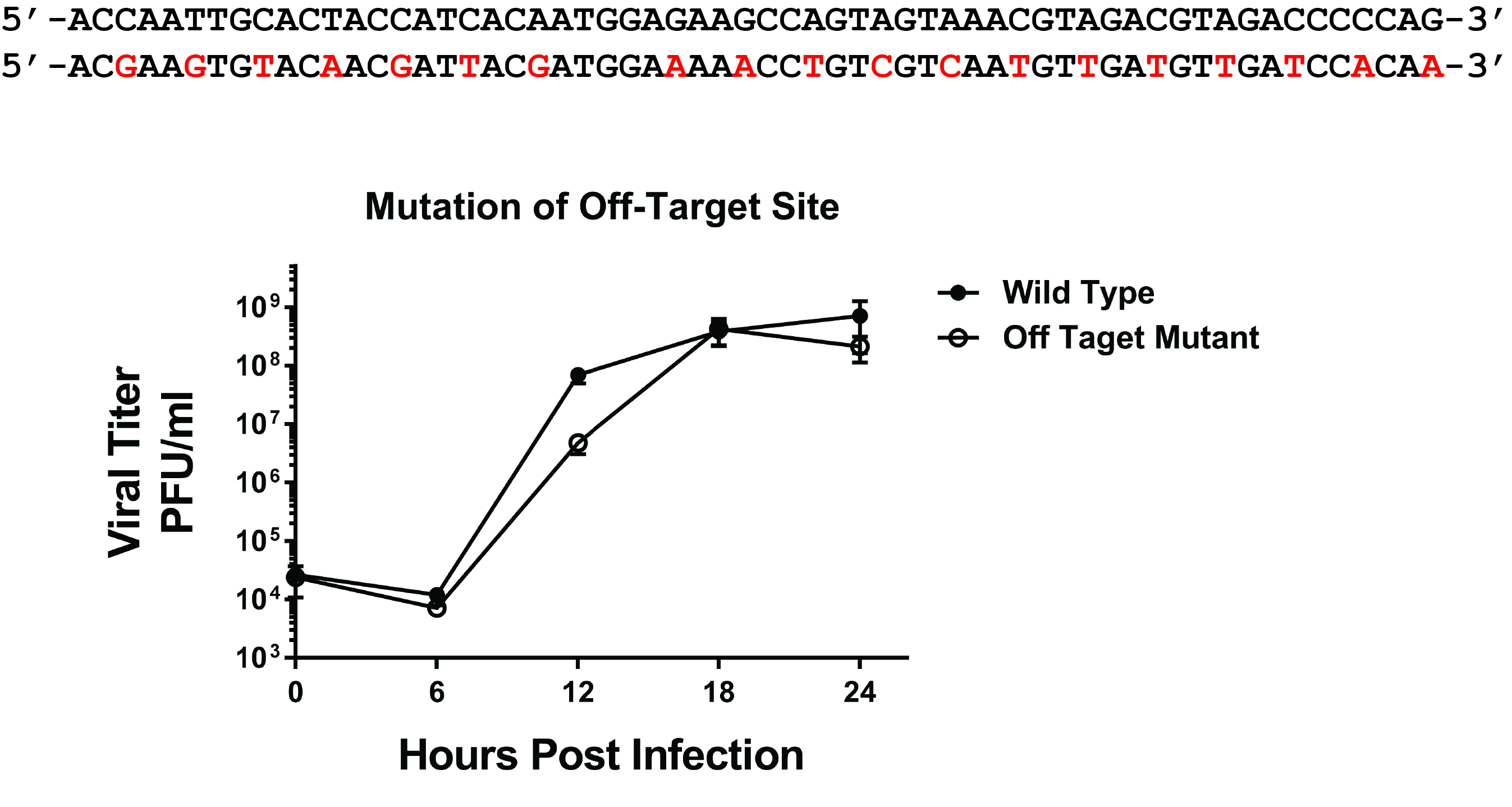

Supplement: S3 Fig — The one-step growth kinetics of parental and a non C:R interaction site mutant as observed in HEK293 cells. Briefly, a region of the SINV genomic RNA detected, but identified as statistically insignificant, by CLIP-seq analysis was mutated using identical parameters to the bona fide C:R interaction site mutants. This region corresponds to nt41-98 of the viral genomic RNA, which includes the genuine start site of nsP1. The quantitative data in this figure represents the mean of three independent biological replicates, the error bar representing the standard deviation of the mean. (TIF) [file ppat.1006473.s005.tif]
